# Patient-centered respectful maternity care: a factor analysis contextualizing marginalized identities, trust, and informed choice

**DOI:** 10.1186/s12884-024-06491-2

**Published:** 2024-04-11

**Authors:** Annie Glover, Carly Holman, Patrick Boise

**Affiliations:** 1 University of Montana Rural Institute for Inclusive Communities, Missoula, US; 2 University of Montana School of Public & Community Health Sciences, Missoula, US

**Keywords:** Maternity care, Patient-centered care, Respectful care, Patient autonomy, Health disparities

## Abstract

**Background:**

Increasing rates of maternal mortality and morbidity, coupled with ever-widening racial health disparities in maternal health outcomes, indicate that radical improvements need to be made in the delivery of maternity care. This study explored the provision of patient-centered maternity care from the perspective of pregnant and postpartum people; experiences of respect and autonomy were examined through the multi-dimensional contexts of identity, relational trust, and protection of informed choices.

**Methods:**

We conducted primary data collection among individuals who experienced a pregnancy in the five years preceding the survey (*N* = 484) using the validated Mothers on Respect Index (MORi) and Mothers Autonomy in Decision Making (MADM) scale. We conducted an exploratory factor analysis (EFA) which produced three factor variables: trust, informed choice, and identity. Using these factor variables as dependent variables, we conducted bivariate and multivariate analysis to examine the relationship between these factor variables and social marginalization, as measured by race, disability, justice-involvement, and other social risk factors, such as food and housing insecurity.

**Results:**

Results of our bivariate and multivariate models generally confirmed our hypothesis that increased social marginalization would be associated with decreased experiences of maternity care that was perceived as respectful and protective of individual autonomy. Most notably, AI/AN individuals, individuals who are disabled, and individuals who had at least one social risk factor were more likely to report experiencing identity-related disrespect and violations of their autonomy.

**Conclusions:**

In light of the findings that emphasize the importance of patient identity in their experience in the healthcare system, patient-centered and respectful maternity care must be provided within a broader social context that recognizes unequal power dynamics between patient and provider, historical trauma, and marginalization. Provider- and facility-level interventions that improve patient experiences and health outcomes will be more effective if they are contextualized and informed by an understanding of how patients’ identities and traumas shape their healthcare experience, health-seeking behaviors, and potential to benefit from clinical interventions and therapies.

**Supplementary Information:**

The online version contains supplementary material available at 10.1186/s12884-024-06491-2.

## Background

While maternal morbidity and mortality have decreased globally, the United States faces a continued crisis with rising rates [1, 2]. The Centers for Disease Control and Prevention (CDC) reported that the maternal mortality rate increased from 23.8 deaths per 100,000 live births in 2020 to 32.9 deaths in 2021 [1]. Black and American Indian Alaska Native (AI/AN) individuals, residents of rural communities, and those in lower socioeconomic groups face significantly higher risks during and after pregnancy than the general population [3–5]. The declining status of maternal health in this country has prompted a deeper investigation into the quality and safety of perinatal care. Research has demonstrated that interactions with providers significantly impact pregnancy and childbirth experiences and, ultimately, health outcomes [6, 7]. Patients have widely reported poor treatment during the perinatal period, including disrespect, mistreatment, and a loss of autonomy – with worse outcomes associated with race, class, gender, and other aspects of identity [8–10]. This study aimed to deepen the field’s understanding of patient-centered care in maternal health from the patients’ perspectives to identify practice improvements that adapt to patient preferences, identities, and need for participation in healthcare decisions.

Mistreatment in maternity care ranges in intensity from a lack of supportive care, discrimination, denial of autonomy, to instances of physical and verbal abuse [11]. In a study conducted by Reed et al. on patients’ experiences of birth trauma, interactions with care providers had the biggest impact on birth trauma over and above non-interpersonal factors such as premature labor and hemorrhage [12]. Participants reported having their knowledge disregarded, feeling pressured into procedures, and instances of physical abuse [12]. Several studies have documented patients’ negative experiences with declining care and requesting options that the provider doesn’t support [7, 13, 14]. These interactions incite feelings of pressure, coercion, and a loss of autonomy [7, 13, 14]. In the Giving Voices to Mother’s US Study, one in six respondents reported facing mistreatment in maternity care, with people of color and those facing social, economic, and health challenges experiencing the highest rates of mistreatment [10]. This builds upon other research, highlighting mistreatment, incivility [15], and inequities in the quality of care for people with marginalized identities [10, 16].

Present-day racial disparities in reproductive health have not arisen in a vacuum; rather, unequal power dynamics and injustice that are rooted in historical racism and oppression have institutionalized these disparities over time [17]. Dr. James Marion Sims, known as the father of gynecology, made advancements in the field by operating on enslaved Black people without their consent and anesthesia [17]. Maternity care sits within a healthcare system shaped by a white, western-based model [18]. This model privileges some (white, middle-upper class) while perpetuating harm to others by not providing a culturally safe and inclusive care environment for all patients [18]. A national study examining racial diversity in Obstetrician and Gynecologist (OBGYN) residency and fellowship programs found that from 2012 to 2018, over half (54.2%) of OBGYN residents were white [19]. Looking at other disciplines, this number increased, with 67.8% of Maternal-Fetal Medicine (MFM) trainees being white and 65.2% of Reproductive Endocrinology and Infertility trainees [19]. In 2018–2019, about 1% (0.9%) of OBGYN residents were AI/AN [19]. The populations that experience the worst maternal health outcomes are not reflected in the obstetric workforce making them the least likely to receive racially concordant care. The effect of racial bias on the experience of care is well-documented in the healthcare system writ large. One study found that half of medical residents surveyed as recently as 2015 endorsed long-disproven myths about biological differences between races impacting medical assessment and care, such as the “thick skin myth,” which falsely holds that black people feel less pain than white people [20].

Positive relationships with healthcare providers centered on trust and respect can be a protective factor for patients navigating medical crises and the healthcare system [21, 22]. Shakibazadeh et al. conducted a systematic review of 67 qualitative studies globally and identified twelve domains of respectful maternity care noting the role of preserving dignity, effective communication, respecting women’s choices, maintaining confidentiality, and care that is free from mistreatment [23]. Patients report greater birth satisfaction and positive experiences when they have clear communication with their providers [6], engage in shared-decision making and informed consent [6, 9], and feel in control [24, 25]. Racial concordance—having a shared racial identity between the patient and provider—has been associated with improved patient experiences [26, 27] and clinical care outcomes [28]. Several other factors have been associated with respectful care, including the location of birth (hospital, home, birth center), provider type, and provider gender [8, 10, 16]. Individuals who gave birth outside a hospital, who worked with a midwife, and who had a female provider reported that they experienced lower levels of mistreatment and higher quality care [10, 16]. Since most pregnant people deliver in a hospital where they may not have the ability to choose the race, gender, or specific specialty of their provider, significant work is needed to universally improve respectful care practices across all settings and provider types.

The Institute of Medicine includes patient-centered care as a core domain of healthcare quality. Patient-centered care involves “providing care that is respectful of and responsive to individual patient preferences, needs, and values and ensuring that patient values guide all clinical decisions [29].” Vedam et al., (2017) & Vedam et al. (2017b), developed the Mothers on Respect Index (MORi) and the Mothers Autonomy in Decision Making scale (MADM) as quality and safety measures that can be used in various healthcare and community settings [8, 9]. Both instruments came out of a person-centered research process, including a community consultation with over 1300 pregnant and postpartum individuals to identify core aspects of respect and decision-making in maternity care settings. MORi gathers information on patients’ comfort levels, willingness to ask questions, and experiences of discrimination, while MADM focuses on patients’ sense of agency and autonomy in making decisions [8, 9].

An emerging body of research has started to operationalize the measurement of respectful care through standardized instruments that not only assess aspects of respect but also do so through the lens of individual identity [8–10]. Including identity further contextualizes patient experiences of respectful care within the social hierarchy of healthcare systems and power differentials between patients and providers [30]. Our study adds a unique contribution by including a focused analysis on the experiences of respect and autonomy across several identity groups that have been underrepresented to date in this type of research, including Indigenous populations, individuals with disabilities, and individuals who experience social risk factors such as food and housing insecurity and justice-involvement. Indigenous communities have experienced a unique history of marginalization and discrimination driven by U.S. policies that followed a multi-century timeline of colonization, genocide, removal in the reservation era, assimilation and boarding schools, and finally the beginnings of recognition and sovereignty in the current era [31–33]. These polices have created significant health inequities —yet limited research in the United States has given attention to Indigenous maternal health experiences and outcomes [4]. The history of disability rights in the U.S. has followed a similar path of injustice, with echoes of the U.S. eugenics and institutionalization policies still reverberating today [34, 35]. Our study sought to build upon the body of knowledge and further refine the field’s understanding of respectful care through factor analysis to measure latent constructs within the general concept of respectful care and ultimately to generate actionable care improvement recommendations that can be implemented by healthcare providers and facilities.

## Methods

### Instrument

The Maternal Healthcare Experiences Survey is a 51-item online survey which included the MORi [8], the MADM [9], and the Health Leads Social Screening Tool [36]. It also includes open-ended questions to gather participant descriptions of perinatal care experiences and a set of sociodemographic questions. We measured respectful care with the 14-item MORi, a valid and reliable tool designed to assess the nature of patient-provider relationships and person-centered care. We measured patient autonomy in decision-making with the 7-item MADM scale, a valid and reliable tool designed to assess the process of decision-making during maternity care. The MORi and MADM scales have been widely implemented to measure maternal healthcare experiences [8, 9]. Open-ended questions gathered further detail on experiences of respect and autonomy. We added additional items to collect information on participant sociodemographic attributes (race/ethnicity, education level, income), social risk, pre-pregnancy wellness visits, and birth location (home, hospital, birth center). We measured social risk with the Health Leads Social Screening Tool [36]. Health Leads includes eight social needs (food insecurity, housing instability, utility needs, financial resource strain, transportation challenges, health literacy, childcare, and social isolation) impacting an individual’s health based on findings from the Institute of Medicine, Centers for Medicare & Medicaid Services, and Health Leads [36]. We measured disability status with the standard set of six disability questions used in the American Community Survey [37]. We created an online survey in REDCap and piloted it with community partners, including individuals meeting the study inclusion criteria to assess for population readability and accessibility. The University of Montana Institutional Review Board approved the study (120 − 22).

### Data collection

Data collection occurred from July 26, 2022 – September 14, 2022. The online survey targeted Montanans who experienced a pregnancy in the last five years. Individuals who had a pregnancy that did not result in a live delivery were eligible to participate in the study. We used convenience and purposive sampling methods. We recruited participants through social media platforms Facebook and Instagram via six custom images. The social media campaign included sponsored posts facilitated by the University of Montana Rural Institute for Inclusive Communities platforms. To further facilitate survey participation from individuals who may have experienced marginalization and may be less likely to participate in research studies, we also sent a postcard to all Montana Women, Infants, and Children (WIC) participants, totaling 8,800.

The original 810 survey responses were screened for study eligibility—individuals in Montana who had experienced a pregnancy within the previous five years. Of the initial 810 survey responses, 105 had non-Montana zip codes, 192 were missing zip code information, and 22 had entries that were not valid zip codes. Additionally, 7 respondents were excluded from the final sample due to missing responses to at least one of the primary instrument items. The remaining 484 observations were retained for analysis.

### Factor analysis

To reduce the 21 items measured by the MORi and MADM scale and to facilitate multivariate analysis of composite measures of maternal experiences of respect and autonomy, we performed an exploratory factor analysis (EFA) using the *factormat* command in Stata 18 [38]. The EFA was performed using principal-component factoring on a polychoric correlation matrix given the ordinal variable structure and a promax oblique rotation to allow for correlation between the latent factors.

The instrument’s 21 maternal healthcare experience items use a 6-point Likert scale, 13 of which are in descending order in the instrument, where a higher ordinal value indicates lower magnitude of autonomy or respect. As an example, for the item “madm_1” a response of “1” indicates they completely agree that the provider asked them about their desired involvement in decision making, and a “6” indicates they completely disagree. In order to align all 21 items in ascending order of magnitude of autonomy or respect, those 13 items’ response scales were inverted. As a result, across all 21 items, a higher ordinal response value corresponds to a greater magnitude of autonomy or respect.

Of the 21 Likert scale variables, 5 display low variability with nearly all participants responding at the extreme end of the scale. Kline suggests skewness > 3.0 and kurtosis > 10.0 as conservative rules of thumb for non-normality [39]. Although no items exceed these thresholds, three items’ responses are highly skewed (Appendix D). We evaluated response distributions to assess whether to retain these items in the analysis. Appendix B shows the distribution of responses for the three items with the least internal variation, though distribution is spread across at least two response values for all three items. Although all three components are highly skewed, responses are not restricted to only the extreme value for any. So, we conclude that these three components have adequate variability to provide information about individuals’ experiences. We used the Kaiser-Meyer-Olkin (KMO) measure of sampling adequacy [40–42] and Bartlet’s [43] test of sphericity to assess intercorrelation and appropriateness of the items for factor analysis. The large KMO of 0.952 and Bartlet’s test of sphericity with a *p* = 0.000 indicate strong intercorrelation between items and that a factor analysis is appropriate. All 21 instrument items were retained for the analysis.

The analysis resulted in three factors which we labeled as: Trust (8 items) Informed-Choice (7 items) and Identity (6 items) explaining 77.5% of the variance. Correlation among the items ranged from $r=0.24$ (morb_2 – madm_2) to $r=0.90$ (morb_2 – morb_1), and correlation among factors ranged from $r=0.49$ (Informed Choice - Identity) to $r=0.70$ (Informed Choice - Trust). The factor loadings and the domain to which they belong are shown in Table 1. We calculated the three factor variables using regression on the rotated factor loadings for use in primary models: trust, identity, and informed choice. The items comprising each of these factor variables are detailed in Appendix F. The three factor variables measure specific dimensions of respectful care and patient autonomy. Specifically, “trust” measures a patient’s relational experience with their provider, including feelings of mutual respect and comfort with clinical decisions. “Identity” measures a patient’s assessment of their care within the context of their membership in a marginalized class, their personality, and their perceived power situation. “Informed choice” measures a patient’s experience with receiving information from their provider about their care as well as their ability to act freely on that information.

**Table 1:** Summary statistics for three factor scores

	No. of items	Mean	Standard Deviation	Skewness	Kurtosis	Cronbach’s α	Eigenvalue	Proportion of variance explained
1. Trust	8	5.623	0.950	-1.202	5.222	0.594	12.958	0.617
2. Informed Choice	7	4.846	1.378	-0.651	2.980	0.663	2.338	0.111
3. Identity	6	5.529	1.185	-1.662	5.665	0.735	0.987	0.047

### Regression analysis

Given the documented relationship between patient social positionality and their experiences in healthcare, we hypothesized that individuals who scored lower on the respectful care and autonomy scales would be more likely to also experience other forms of social disempowerment and marginalization. These constructs are measured in this study as: membership in a minoritized racial group, gender marginalization, disability, low socioeconomic status, lower educational attainment, and experience with a social risk factor. To study the association between respondent characteristics and their experiences of the three dimensions of respectful care and autonomy that arose from our factor analysis, we constructed bivariate analyses using t-testing to assess for significant associations between each of the factor variables and respondent characteristics. Three OLS multivariate regression models were constructed to adjust for confounding variables. Covariates were selected for each model if they were significantly associated with the dependent variable in bivariate analysis. The equations below further detail these models; the models adjust for potential confounding using the following covariates: race, education, household income, age, justice-involvement, disabilities, and social risk factors. The factor scores were squared to improve normality of their left-skewed distributions.

$$\eqalign{{Score}_{i}^{2} & = {\beta }_{0}+{\beta }_{1}Race+{\beta }_{2}Edu+{\beta }_{3}HHI+{\beta }_{4}Age \cr & +{\beta }_{5}Justice+{\beta }_{6}Disability+{\beta }_{7}Risk+\epsilon }$$

Where ${Score}_{i}^{2}$ is the squared factor score of the ${i}^{th}$ factors 1:3: Trust, Informed Choice, and Identity,

$$Race=\left\{\begin{array}{cc}1& if\, indicated\, AI/AN\\ 0& otherwise\end{array}\right\},$$

$$Edu=\left\{\begin{array}{cc}1& if\, attained\, high\, school diploma\, at\, most\\ 0& otherwise\end{array}\right\},$$

$$HHI=\left\{\begin{array}{cc}1& if\, houshold income<\$\text{75,000}\\ 0& otherwise\end{array}\right\},$$

$$Age=\left\{\begin{array}{cc}1& if\, age\, 29\, or\, younger\\ 0& otherwise\end{array}\right\},$$

$$Justice=\left\{\begin{array}{cc}1& if\, self\, or\, other\, parent\, ever\, incarcerated\\ 0& otherwise\end{array}\right\},$$

$$Disability=\left\{\begin{array}{cc}1& if\, indicated\, one\, or more\, disability\\ 0& otherwise\end{array}\right\},$$

$${\rm and }\, Risk=\left\{\begin{array}{cc}1& if\, one\, or more\, social\, risk\, factor\, indicated\\ 0& otherwise\end{array}\right\}.$$

These variables were collapsed from the original multi-level categorical variables for more extensive measurement of effect on factor scores. Each has been reduced to an indicator variable of participant exposure to social disempowerment, including being younger, having a disability, social risk factor, lower income, or lower educational attainment. Each is coded as a new indicator variable with outcomes that are mutually exclusive and exhaustive.

## Results

Table 2 below provides detailed descriptive statistics of the study population (*N* = 484). While this is a convenience rather than probability sample and cannot be statistically generalized to the population, the study sample does indicate frequency matching to Montana at large, across several key demographic characteristics. While most of the sample (88.4%) were white, the AI/AN community comprises approximately 6% of Montana’s population. Over half (54.3%) of survey respondents live in rural counties. Montana’s median household income in 2022 was $72,980; less than half (40.8%) of respondents reported an annual household income of $75,000 or more. According to the CDC, 28% of Montanans across all age groups have a disability; in this sample of younger people, 20.9% reported having a disability [44].

**Table 2:** Select demographic descriptive statistic

*N* = 484	n (%)
**Race/ethnicity** (8.9% selected more than 1)	
American Indian, Native American, Alaska Native	41 (8.5%)
African, African American, or Black	9 (1.9%)
Asian or Asian American	9 (1.9%)
Hispanic/Latinx	26 (5.4%)
Middle Eastern or North African	3 (0.6%)
Native Hawaiian or Pacific Islander	3 (0.6%)
White	428 (88.4%)
Prefer not to answer	12 (2.5%)
**Gender**	
Woman	469 (96.9%)
Other Gender Identity*	11 (2.3%)
Prefer not to answer	4 (0.8%)
**Age range**	
18–29	137 (28.3%)
30–39	296 (61.2%)
>=40	51 (10.5%)
**Educational Attainment**	
High School or less	58 (12.0%)
Some College	152 (31.4%)
Bachelor’s	155 (32.0%)
Graduate Degree/Professional	119 (24.6%)
**Labor force attachment**	
Labor Force Employed	354 (73.1%)
Homemaker or Student	95 (19.6%)
Unemployed Seeking	15 (3.1%)
Non-Labor Force or Other	20 (4.1%)
**Annual household Income**	
$0–24,999	59 (12.2%)
$25,000–49,999	115 (23.8%)
$50,000–74,999	112 (23.2%)
$75,000–99,999	73 (15.1%)
>$100,000	124 (25.7%)
Rurality by RUCA* **designation**	
Urban: Metropolitan Core	183 (37.8%)
Urban: Metropolitan high commuting	38 (7.9%)
Rural: Micropolitan Core	113 (23.3%)
Rural: Micropolitan high commuting	4 (0.8%)
Rural: Small town core	74 (15.3%)
Rural: Small town high commuting	4 (0.8%)
Rural areas	68 (14.0%)
**Have you or your child’s other parent ever been incarcerated?**	
Yes, I have	6 (1.2%)
Yes, my child’s second parent has	30 (6.2%)
Yes, my child’s second parent and I have both been incarcerated	11 (2.3%)
No	435 (90.2%)
**Disability Status** (8.5% selected more than 1)	
Are you deaf, or do you have serious difficulty hearing?	10 (2.1%)
Are you blind, or do you have serious difficulty seeing, even when wearing glasses?	11 (2.3%)
Because of a physical, mental, or emotional condition, do you have serious difficulty concentrating, remembering, or making decisions?	79 (16.4%)
Do you have serious difficulty walking or climbing stairs?	17 (3.5%)
Do you have difficulty dressing or bathing?	11 (2.3%)
Because of a physical, mental, or emotional condition, do you have difficulty doing errands alone such as visiting a doctor’s office or shopping?	44 (9.1%)

*Other gender includes self-identification to one of: Genderqueer/gender-nonconforming neither exclusively male nor female; Man; Transgender man/trans man; Transgender woman/trans woman; Two-Spirit; Something else; Prefer not to answer

*Rural Urban Commuting Area (RUCA) are developed by the USDA as a measure of population density, and areas near urban centers where a significant proportion of the population commutes to an urban area [45]. These codes range from 1 to 10, with higher values indicating lower population density. We define rural areas as RUCA codes 3–10, where at most, fewer than 30% of the population flows to urban areas. And urban is defined as RUCA codes 1 and 2, where at least 30% of the population flows to urban areas, or resides in an urban area (“Metropolitan core” and “Metropolitan high commuting”)

Table 3 provides detailed descriptive statistics of relevant contextual factors that illuminate the healthcare experiences reported by survey respondents. Just over half (63.0%) of respondents reported that they had an annual visit with a physician in the year preceding their pregnancy, despite the Women’s Preventative Services Initiative (WPSI) universal recommendation for annual well-woman care. Nearly 1 out of 6 (16.5%) respondents reported experiencing food insecurity, and half (50.1%) reported childcare challenges. About 1 out of 10 respondents reported some form of housing insecurity, with 9.5% reporting delinquency in paying housing-related utilities and 9.3% experiencing instability in their housing.

**Table 3:** Contextual descriptive statistics

*N* = 484	n (%)
**Providers involved in the 12 months following most recent pregnancy** (65.9% selected more than 1)	
Family Doctor/Primary Care Provider	169 (34.9%)
Certified Nurse Midwife	133 (27.5%)
Direct Entry Midwife / Lay Midwife	40 (8.3%)
Nurse Practitioner (NP)	115 (23.8%)
Obstetrician/Gynecologist (OB/GYN)	363 (75.0%)
Maternal-Fetal Medicine (MFM) Specialist/Perinatologist	130 (26.9%)
Physician Assistant/Physician Associate (PA)	70 (14.5%)
Other	28 (5.8%)
**Where delivery took place**	
Hospital	373 (83.1%)
At home	27 (6.0%)
Birth center	29 (6.5%)
Other (please specify):	4 (0.9%)
I did not deliver (early pregnancy loss, miscarriage, termination)	16 (3.6%)
**Did you have an annual wellness visit the year before your pregnancy?**	
Yes	305 (63.0%)
No	154 (31.8%)
Unsure	25 (5.2%)
**Were you satisfied with the care you received at your wellness visit?**	
Yes	297 (97.4%)
No	8 (2.6%)
**Social Risk Factors** (32.2% selected more than 1)	
In the last 12 months, did you eat less than you felt you should because there wasn’t enough money for food?	80 (16.5%)
In the last 12 months, has the electric, gas, oil, or water company threatened to shut off services in your home?	46 (9.5%)
Are you worried that in the next 2 months, you may not have stable housing?	45 (9.3%)
Do problems getting child care make it difficult for you to work or study?	226 (50.1%)
In the last 12 months, have you needed to see a doctor, but could not because of cost?	63 (13.0%)
In the last 12 months, have you ever had to go without health care because you didn’t have a way to get there?	31 (6.4%)
Do you ever need help reading hospital materials?	31 (6.4%)
Do you often feel that you lack companionship?	118 (24.4%)

As described above, three factor variables were created to reduce the 21 items measured in the respect and autonomy instruments to composite domains of patient experiences. The summary statistics of these factor scores are provided in Table 1. The lowest scores reported were for those items included in the “informed choice” factor variable.

To assess the association between the three factor domains of patient experience of respectful care and relevant experience, we conducted bivariate analysis using t-tests. The results of the bivariate analyses are detailed below in Table 4, with statistical significance identified for those associations with a *P* < 0.05. We found statistically significant differences across several sociodemographic attributes as follows. AI/AN respondents were less likely to report that they experienced a trusting relationship with their healthcare provider during pregnancy than respondents who did not identify as AI/AN, and they were also less likely to report experiencing identity-related respectful care. White respondents were more likely than non-White respondents to report experiencing identity-related respectful care. Individuals reporting that they had one or more disabilities were less likely to report experiencing identity-related respectful care than individuals who are not disabled. Individuals who reported at least one social risk factor were also less likely to feel that they had a trusting relationship with their provider or that their informed choice was protected during their reproductive health care experiences. Justice involvement was also associated with lower levels of respectful care in the trust and identity dimensions. In a few instances, increased social marginalization was not associated with lower levels of respectful care, as hypothesized. Individuals who had a high school diploma or less as well as younger individuals were more likely to report trusting their healthcare provider.

**Table 4:** Bivariate tables of factor score by select characteristics

		1.Trust	2.Informed Choice	3.Identity
**Race: AI/AN**	**n (%)**	**Mean (sd)**	**Mean (sd)**	**Mean (sd)**
AI/AN	41 (8.5%)	5.280 (1.358)	4.594 (1.359)	5.162 (1.164)
Not AI/AN	443 (91.5%)	5.654 (0.898)	4.870 (1.379)	5.562 (1.183)
		*p* = 0.015	*p* = 0.221	*p* = 0.038
**Race: White**	**n(%)**	**Mean (sd)**	**Mean (sd)**	**Mean (sd)**
White	428 (88.4%)	5.643 (0.952)	4.864 (1.386)	5.616 (1.137)
Not White	56 (11.6%)	5.464 (0.922)	4.710 (1.323)	4.857 (1.335)
		*p* = 0.183	*p* = 0.431	p = < 0.001
**Race: Other**	**n(%)**	**Mean (sd)**	**Mean (sd)**	**Mean (sd)**
Other*	59 (12.2%)	5.604 (0.964)	4.820 (1.383)	5.112 (1.312)
Not Other	425 (87.8%)	5.625 (0.949)	4.850 (1.379)	5.586 (1.156)
		*p* = 0.874	*p* = 0.874	*p* = 0.004
**RUCA Classification**	**n(%)**	**Mean (sd)**	**Mean (sd)**	**Mean (sd)**
Rural areas	68 (14.0%)	5.638 (0.673)	5.430 (0.384)	5.366 (1.246)
Small town high commuting	4 (0.8%)	5.638 (0.673)	5.430 (0.384)	6.517 (0.157)
Small town core	74 (15.3%)	5.447 (1.172)	4.614 (1.545)	5.363 (1.202)
Micropolitan high commuting	4 (0.8%)	6.111 (0.470)	5.188 (1.135)	4.649 (2.739)
Micropolitan Core	113 (23.3%)	5.662 (0.895)	4.839 (1.374)	5.627 (1.110)
Metropolitan high commuting	38 (7.9%)	5.582 (1.090)	4.867 (1.373)	5.313 (1.480)
Metropolitan Core	183 (37.8%)	5.737 (0.829)	4.954 (1.321)	5.637 (1.078)
		*p* = 0.162	*p* = 0.61	*p* = 0.079
**Educational Attainment**	**n(%)**	**Mean (sd)**	**Mean (sd)**	**Mean (sd)**
High School or less	58 (12.0%)	5.833 (0.903)	5.275 (1.334)	5.785 (0.965)
Some College	152 (31.4%)	5.470 (1.128)	4.731 (1.480)	5.263 (1.338)
Bachelor’s	155 (32.0%)	5.595 (0.850)	4.782 (1.359)	5.544 (1.184)
Graduate Degree/Professional	119 (24.6%)	5.751 (0.810)	4.868 (1.261)	5.722 (1.011)
		*p* = 0.027	*p* = 0.071	*p* = 0.003
**Employment**	**n(%)**	**Mean (sd)**	**Mean (sd)**	**Mean (sd)**
Non-Labor Force or Other	20 (4.1%)	5.581 (1.030)	5.152 (1.124)	5.447 (1.299)
Unemployed Seeking	15 (3.1%)	5.337 (1.010)	4.514 (1.102)	4.958 (1.339)
Homemaker or Student	95 (19.6%)	5.670 (1.110)	4.953 (1.580)	5.681 (1.085)
Labor Force Employed	354 (73.1%)	5.624 (0.896)	4.814 (1.343)	5.516 (1.194)
		*p* = 0.653	*p* = 0.455	*p* = 0.159
**Annual Household Income**	**n(%)**	**Mean (sd)**	**Mean (sd)**	**Mean (sd)**
$0–24,999	59 (12.2%)	5.787 (0.951)	5.307 (1.223)	5.688 (1.136)
$25,000–49,999	115 (23.8%)	5.609 (1.015)	4.823 (1.554)	5.428 (1.266)
$50,000–74,999	112 (23.2%)	5.400 (1.008)	4.661 (1.420)	5.134 (1.425)
$75,000–99,999	73 (15.1%)	5.752 (0.764)	4.975 (1.227)	5.719 (1.084)
>$100,000	124 (25.7%)	5.675 (0.907)	4.726 (1.277)	5.782 (0.802)
		*p* = 0.045	*p* = 0.036	p = < 0.001
**Age**	**n(%)**	**Mean (sd)**	**Mean (sd)**	**Mean (sd)**
18–29	137 (28.3%)	5.805 (0.878)	4.972 (1.458)	5.795 (0.859)
30–39	296 (61.2%)	5.561 (0.977)	4.809 (1.361)	5.424 (1.300)
>=40	51 (10.5%)	5.491 (0.924)	4.724 (1.253)	5.421 (1.149)
		*p* = 0.026	*p* = 0.417	*p* = 0.008
**Justice Involved**	**n(%)**	**Mean (sd)**	**Mean (sd)**	**Mean (sd)**
Yes, I have	6 (1.2%)	4.740 (1.073)	4.507 (0.598)	4.264 (1.567)
Yes, my child’s second parent has	30 (6.2%)	5.512 (0.970)	5.034 (1.385)	5.393 (1.360)
Yes, my child’s second parent and I have both been incarcerated	11 (2.3%)	4.717 (1.216)	4.655 (1.136)	4.620 (1.281)
No	435 (90.2%)	5.664 (0.927)	4.838 (1.393)	5.575 (1.151)
		p = < 0.001	*p* = 0.769	*p* = 0.002
**Disabilities**				
Are you deaf, or do you have serious difficulty hearing?	**n(%)**	**Mean (sd)**	**Mean (sd)**	**Mean (sd)**
Yes	10 (2.1%)	5.201 (0.934)	4.864 (1.225)	4.644 (1.713)
No	474 (97.9%)	5.631 (0.949)	4.846 (1.383)	5.547 (1.167)
		*p* = 0.156	*p* = 0.968	*p* = 0.017
Are you blind, or do you have serious difficulty seeing, even when wearing glass	**n(%)**	**Mean (sd)**	**Mean (sd)**	**Mean (sd)**
Yes	11 (2.3%)	4.976 (1.259)	4.389 (1.294)	4.685 (1.359)
No	472 (97.7%)	5.641 (0.935)	4.859 (1.381)	5.552 (1.173)
		*p* = 0.021	*p* = 0.265	*p* = 0.016
Because of a physical, mental, or emotional condition, do you have serious s difficulty concentrating, remembering, or making decisions?	**n(%)**	**Mean (sd)**	**Mean (sd)**	**Mean (sd)**
Yes	79 (16.4%)	5.692 (1.049)	5.031 (1.440)	5.280 (1.347)
No	404 (83.6%)	5.609 (0.931)	4.812 (1.366)	5.577 (1.148)
		*p* = 0.48	*p* = 0.198	*p* = 0.042
Do you have serious difficulty walking or climbing stairs? Or difficulty dressing or bathing?	**n(%)**	**Mean (sd)**	**Mean (sd)**	**Mean (sd)**
Yes	20 (4.1%)	5.099 (1.254)	4.753 (1.399)	4.659 (1.503)
No	464 (95.9%)	5.645 (0.929)	4.850 (1.379)	5.566 (1.157)
		*p* = 0.012	*p* = 0.757	p = < 0.001
Because of a physical, mental, or emotional condition, do you have difficulty doing errands alone such as visiting a doctor’s office or shopping?	**n(%)**	**Mean (sd)**	**Mean (sd)**	**Mean (sd)**
Yes	44 (9.1%)	5.132 (1.147)	4.493 (1.502)	4.789 (1.357)
No	439 (90.9%)	5.670 (0.915)	4.878 (1.361)	5.601 (1.143)
		p = < 0.001	*p* = 0.077	p = < 0.001
**Social Risk Factors**				
In the last 12 months, did you ever eat less than you felt you should because there wasn’t enough money for food?	**n(%)**	**Mean (sd)**	**Mean (sd)**	**Mean (sd)**
Yes	80 (16.5%)	5.506 (1.141)	4.861 (1.591)	5.170 (1.337)
No	404 (83.5%)	5.646 (0.907)	4.843 (1.335)	5.600 (1.141)
		*p* = 0.23	*p* = 0.918	*p* = 0.003
In the last 12 months, has the electric, gas, oil, or water company threatened to shut off services in your home?	**n(%)**	**Mean (sd)**	**Mean (sd)**	**Mean (sd)**
Yes	46 (9.5%)	5.376 (1.230)	4.377 (1.615)	5.078 (1.337)
No	437 (90.5%)	5.649 (0.914)	4.895 (1.345)	5.575 (1.161)
		*p* = 0.064	*p* = 0.015	*p* = 0.007
Are you worried that in the next 2 months, you may not have stable housing?	**n(%)**	**Mean (sd)**	**Mean (sd)**	**Mean (sd)**
Yes	45 (9.3%)	5.250 (1.203)	4.546 (1.525)	4.863 (1.287)
No	439 (90.7%)	5.661 (0.913)	4.877 (1.361)	5.597 (1.154)
		*p* = 0.006	*p* = 0.125	p = < 0.001
Do problems getting child care make it difficult for you to work or study?	**n(%)**	**Mean (sd)**	**Mean (sd)**	**Mean (sd)**
Yes	226 (50.1%)	5.485 (1.029)	4.607 (1.434)	5.486 (1.096)
No	225 (49.9%)	5.732 (0.854)	5.082 (1.260)	5.518 (1.316)
		*p* = 0.006	p = < 0.001	*p* = 0.78
In the last 12 months, have you needed to see a doctor, but could not because of cost?	**n(%)**	**Mean (sd)**	**Mean (sd)**	**Mean (sd)**
Yes	63 (13.0%)	5.246 (1.100)	4.168 (1.590)	5.016 (1.322)
No	420 (87.0%)	5.679 (0.914)	4.950 (1.317)	5.606 (1.147)
		p = < 0.001	p = < 0.001	p = < 0.001
In the last 12 months, have you ever had to go without health because you didn’t have a way to get there?	**n(%)**	**Mean (sd)**	**Mean (sd)**	**Mean (sd)**
Yes	31 (6.4%)	5.126 (1.094)	4.245 (1.499)	4.311 (1.336)
No	453 (93.6%)	5.657 (0.931)	4.888 (1.362)	5.612 (1.129)
		*p* = 0.003	*p* = 0.012	p = < 0.001
Do you ever need help reading hospital materials?	**n(%)**	**Mean (sd)**	**Mean (sd)**	**Mean (sd)**
Yes	31 (6.4%)	5.003 (0.908)	4.534 (1.162)	4.274 (1.294)
No	452 (93.6%)	5.665 (0.939)	4.867 (1.392)	5.613 (1.130)
		p = < 0.001	*p* = 0.194	p = < 0.001
Do you often feel that you lack companionship?	**n(%)**	**Mean (sd)**	**Mean (sd)**	**Mean (sd)**
Yes	118 (24.4%)	5.304 (1.049)	4.330 (1.456)	5.143 (1.213)
No	365 (75.6%)	5.730 (0.888)	5.015 (1.313)	5.659 (1.146)
		p = < 0.001	p = < 0.001	p = < 0.001

*Other race/ethnicity includes self-identification of any/all: African, African American, or Black; Asian or Asian American; Hispanic/Latinx; Middle Eastern or North African; Native Hawaiian or Pacific Islander; Something else; Prefer not to answer

As described above, the bivariate analysis demonstrated several significant relationships between participant characteristics related to social status and marginalization and our factor variables measuring the three dimensions of respectful care. Table 5 provides the results from our multivariate regression analysis. Coefficients reported in this linear regression represent the squared difference in the mean score of each of the factor variables for that subgroup compared to the reference category. For example, an individual who is AI/AN is predicted to have an adjusted mean score for respectful and autonomous care in the identity domain of $\sqrt{3.732}$ less than individuals who are not AI/AN. However, since these scores do not have tangible units of measure, we present interpretations of the coefficients here based on directionality and qualitative magnitude. Coefficients that are not statistically significant should be interpreted as having no association and are not presented in this narrative.

**Table 5:** OLS regression of factor score on demographic characteristics

	(1)	(2)	(3)
	Trust $$\varvec{\beta }$$ $$\left(\varvec{s}\varvec{e}\right)$$	Informed Choice $$\varvec{\beta }$$ $$\left(\varvec{s}\varvec{e}\right)$$	Identity $$\varvec{\beta }$$ $$\left(\varvec{s}\varvec{e}\right)$$
**Race**			
Not AI/AN	ref.	ref.	ref.
AI/AN	-2.461 (1.617)	-3.015 (2.074)	-3.732^*^ (1.802)
**Education**			
At least some college	ref.	ref.	ref.
High School or less	2.418 (1.451)	3.482 (1.861)	3.074 (1.617)
**Household Income**			
HHI ≥ $75,000	ref.	ref.	ref.
HHI less than $75k	-1.393 (0.948)	0.690 (1.217)	-3.672^***^ (1.057)
**Age**			
30 or older	ref.	ref.	ref.
18–29 y/o	2.637^*^ (1.022)	1.322 (1.311)	4.014^***^ (1.139)
**Justice-involved**			
No	ref.	ref.	ref.
Self or other parent has been incarcerated	-3.544^*^ (1.507)	0.329 (1.934)	-2.561 (1.680)
**Disability**			
No disabilities indicated	ref.	ref.	ref.
One or More Disabilities	1.397 (1.135)	2.384 (1.456)	-2.609^*^ (1.265)
**Social Risk Factors**			
No social risk factors	ref.	ref.	ref.
One or more social risk factors	-2.750^**^ (0.950)	-4.263^***^ (1.218)	-0.738 (1.058)
Constant	34.33^***^ (0.855)	26.61^***^ (1.097)	34.17^***^ (0.953)
Observations	447	447	447
R2	0.071	0.050	0.091
Adjusted R2	0.058	0.036	0.077

^*^p < 0.05, ^**^p < 0.01, ^***^p < 0.001

Across the trust domain, participants reported experiencing lower levels of respectful maternity care if they or their child’s other parent was justice-involved and if they reported having one or more social risk factors (such as having experienced housing or food insecurity in the last year). Notably, the experience of receiving respectful maternity care related to participant identity was the most dramatically impacted. In the multivariate model, individuals identifying as AI/AN, those with a household income lower than $75,000 (approximating Montana’s 2022 median household income), and those with one or more disabilities reported that they were less likely to experience identity-related respectful maternal healthcare.

## Discussion

As respectful maternity care gains momentum as a core strategy for improving maternal health, more must be done to understand respectful care in practice and advance quality improvement initiatives aimed at respectful patient-provider interactions. Our study results point toward several aspects of identity associated with lower levels of respectful care, including patient characteristics related to social status and marginalization. These findings align with recent studies implementing the MORi and MADM scale in the United States and internationally. Several studies found lower levels of respect among women with social risk factors [8, 46, 47]. Almanza et al. also observed lower levels of respect among racial/ethnic minorities [26]. Our study supports the extensive body of literature demonstrating that people with marginalized identities experience significant differences in the quality of care. While we focused on patient-provider interactions, we must position our results within the broader health system to understand the impact of hierarchical structures on interpersonal dynamics.

The Socio-ecological model provides a helpful framework for understanding the complex interplay of individual and environmental factors that shape health outcomes at the levels of intrapersonal factors, interpersonal processes, institutional factors, community factors, and public policy [48]. While much attention has centered on policy efforts to address inequitable economic and social conditions, they must be coupled with institutional/organizational initiatives and interpersonal processes to improve the delivery of quality care, particularly for marginalized groups [49]. The multidimensional experience of respectful care highlights the inherent relational nature of respectful maternity care, shaped by ongoing interactions between the patient and provider. Respectful maternity care hinges on providers awareness of these dynamics and ability to prioritize patient identities and preferences over personal beliefs and health system constraints [50].

This study contributes a nuanced measure of respectful maternity care through the use of exploratory factor analysis. By reducing the 21-items from the MADM and MORi scales into three composite factor variables, researchers and clinicians have a multidimensional view of the lived experience of respectful maternity care, particularly within the sociodemographic and historical context of the American healthcare system. Bioethicists have long understood that the pillars of bioethics—justice, beneficence, nonmaleficence, and informed consent—must be protected as a cohesive unit, rather than four disconnected parts, to safeguard patients and research participants. Likewise, patients who report experiences of disrespectful care may feel that they have been mistreated in myriad ways; they may not feel that they can trust their provider, that their provider is giving them the information they need to make an autonomous and free choice in their care, or that they are being treated poorly due to their identity. This study enriches and contributes much-needed specificity to respectful care guidelines; providers seeking to improve the care they are offering to their patients should employ relational strategies that (1) build trust with their patients; (2) communicate for understanding; (3) promote patient engagement in healthcare decision-making; and (4) ensure the cultural safety and inclusivity of their practice and healthcare setting.

While this study provides a significant contribution to the field of patient-centered care, it does have limitations. First, feelings and perceptions that individuals have as they move through the world and the healthcare system cannot be universally standardized to objective mathematical models that attempt to quantify experiences. The experience of respectful care is highly subjective, and the perception of that experience is shaped by multiple intersecting identities and contexts. However, these models do offer some important insight. Given that the multivariate models serve to adjust predictions around a standard set of covariates, these coefficients represent the independent effects of race, socioeconomic status, and disability on the experience of respectful care. Individuals who are members of a minoritized racial group are less likely to experience identity-related respectful care even if they are in a higher income bracket; socioeconomic status does not account for racial, disability-status, or other identity-related disparities in the experience of care.

Another limitation of this study is the convenience sample that was utilized for the study. While this sample lacks external validity—conclusions cannot be inferred to the general population—the use of the validated scales, intensive piloting of the survey, and the purposive sampling increased internal validity. The constructs measured by this sample have solid backing in the literature, and the lessons generated through this study are transferrable to clinical practice improvements. Ultimately, despite these limitations, this study provides actionable steps that can inform provider- and facility-level interventions that can have a material benefit to pregnant patients. Future research should expand upon and improve the inquiry that we initiated here by authentically employing Indigenous research methodologies and community-based participatory research that can further refine and generate meaningful recommendations that will deconstruct societal power hierarchies that are replicated within healthcare settings and are not serving patients well.

## Conclusions

The results from this study deepen the field’s understanding of respectful care—a practice that must be individualized to patients’ backgrounds and identities. Integrating the MORi and MADM scale within facilities as quality and safety measures and emphasizing informed choice, patient preferences, and patient identities in care delivery can support continuous improvement and ensure equity. Efforts must prioritize the quality of care for historically marginalized groups to address the stark disparities in maternal health experiences and outcomes in the United States.

### Electronic supplementary material

Below is the link to the electronic supplementary material.

Supplementary Material 1

Supplementary Material 2

## Data Availability

The datasets used and/or analyzed during the current study are available from the corresponding author upon reasonable request.

## Abbreviations

AI/AN: American Indian Alaska Native
CDC: Centers for Disease Control and Prevention
EFA: Exploratory Factor Analysis
KMO: Kaiser-Meyer-Olkin
MORi: Mothers on Respect Index
MADM: Mothers Autonomy in Decision Making Scale
OBGYN: Obstetrician and Gynecologist
OLS: Ordinary least-squares
RUCA: Rural Urban Commuting Area
WIC: Special Supplemental Nutrition Program for Women, Infants, and Children
WPSI: Women’s Preventative Services Initiative
