# Supplementary material for: Patient-centered respectful maternity care: a factor analysis contextualizing marginalized identities, trust, and informed choice
Source: BMC Pregnancy Childbirth. 2024 Apr 11;24:267. doi: 10.1186/s12884-024-06491-2 (PMC11010273; doi:10.1186/s12884-024-06491-2)
Supplement: Supplementary file 1 — Supplementary Material 1 [file 12884_2024_6491_MOESM1_ESM.docx]

Appendix

**A.** Assessment of distribution normality

| **Variable** | **Mean** | **SD** | **Skewness** | **Kurtosis** |
| --- | --- | --- | --- | --- |
| madm_1 | 4.450413 | 1.537529 | -0.80803 | 2.657847 |
| madm_2 | 4.376033 | 1.413893 | -0.59172 | 2.624228 |
| madm_3 | 4.466942 | 1.372965 | -0.65881 | 2.673226 |
| madm_4 | 4.869835 | 1.195921 | -1.02774 | 3.670561 |
| madm_5 | 4.613636 | 1.313058 | -0.77253 | 2.903569 |
| madm_6 | 4.826446 | 1.223833 | -1.0434 | 3.730946 |
| madm_7 | 5.095041 | 1.082161 | -1.37651 | 4.912516 |
| mor_1 | 5.42562 | 0.831973 | -1.85777 | 7.679976 |
| mor_2 | 4.729339 | 1.260602 | -0.85268 | 3.009169 |
| mor_3 | 5.196281 | 0.969892 | -1.62693 | 6.455842 |
| mor_4 | 4.31405 | 1.483005 | -0.61371 | 2.305003 |
| mor_5 | 4.97314 | 0.980821 | -0.89519 | 3.791294 |
| mor_6 | 5.214876 | 0.981878 | -1.46467 | 5.189617 |
| mor_7 | 5.303719 | 0.856534 | -1.77252 | 7.445696 |
| morb_1 | 5.35124 | 1.212674 | -2.16793 | 6.966478 |
| morb_2 | 5.495868 | 1.040576 | -2.57706 | 9.605029 |
| morb_3 | 5.336777 | 1.191851 | -2.05252 | 6.619895 |
| morb_4 | 5.076446 | 1.303103 | -1.40617 | 4.053211 |
| morc_1 | 4.22314 | 1.59184 | -0.47614 | 2.044743 |
| morc_2 | 4.747934 | 1.356869 | -0.9058 | 2.772802 |
| morc_3 | 4.43595 | 1.558397 | -0.59537 | 2.061918 |

**B.**
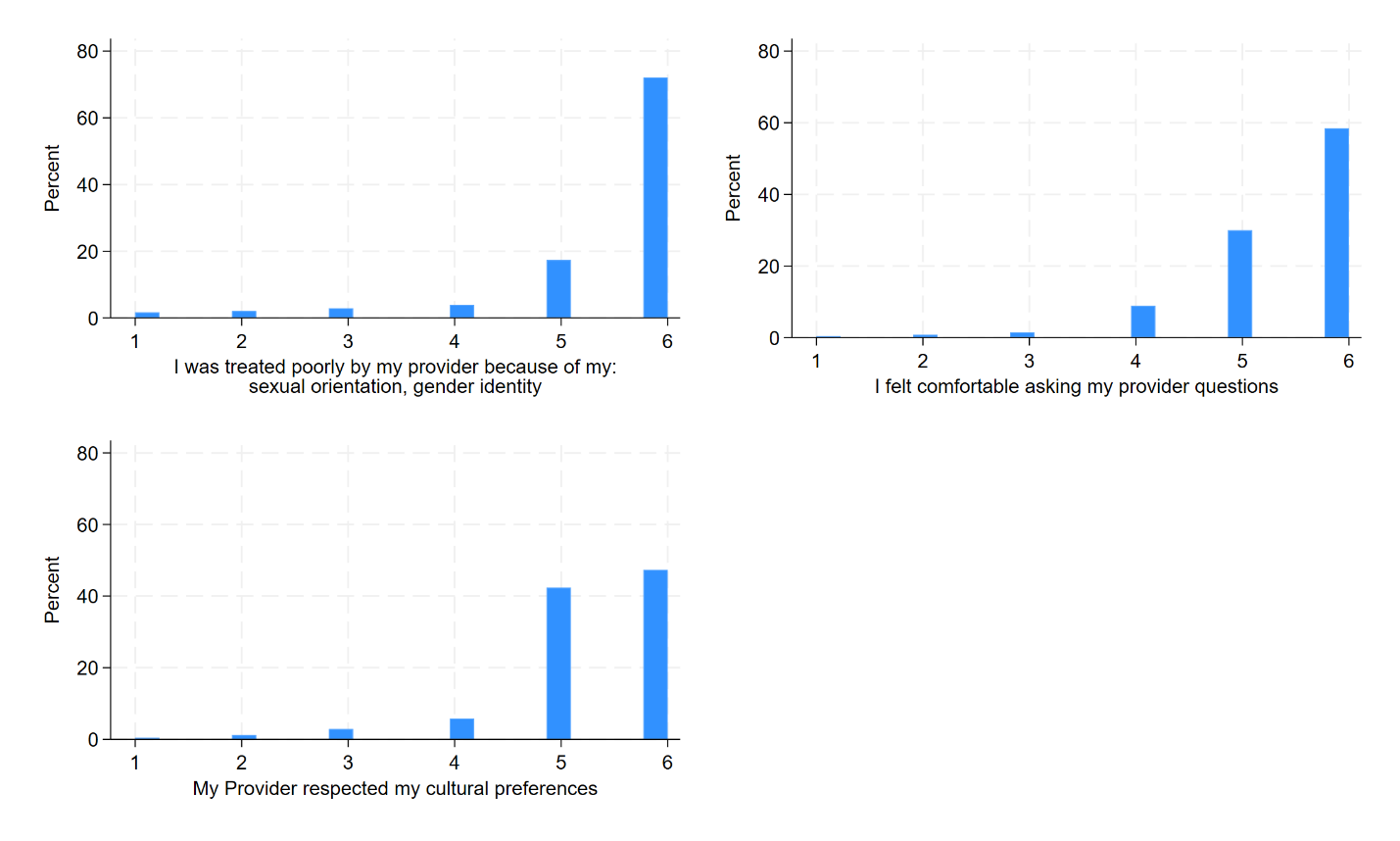


**C.** Item testing for adequacy.

| Determinant of the correlation matrix |  |
| --- | --- |
| Det = 0.000 |  |
|  |  |
| Bartlett test of sphericity |  |
| Chi-square = 8300.970 |  |
| Degrees of freedom = 210 |  |
| p-value = 0.000 |  |
| H0: variables are not intercorrelated |  |
|  |  |
| Kaiser-Meyer-Olkin Measure of Sampling | Adequacy |
| KMO = 0.952 |  |

**D.** Polychoric correlation matrix of items


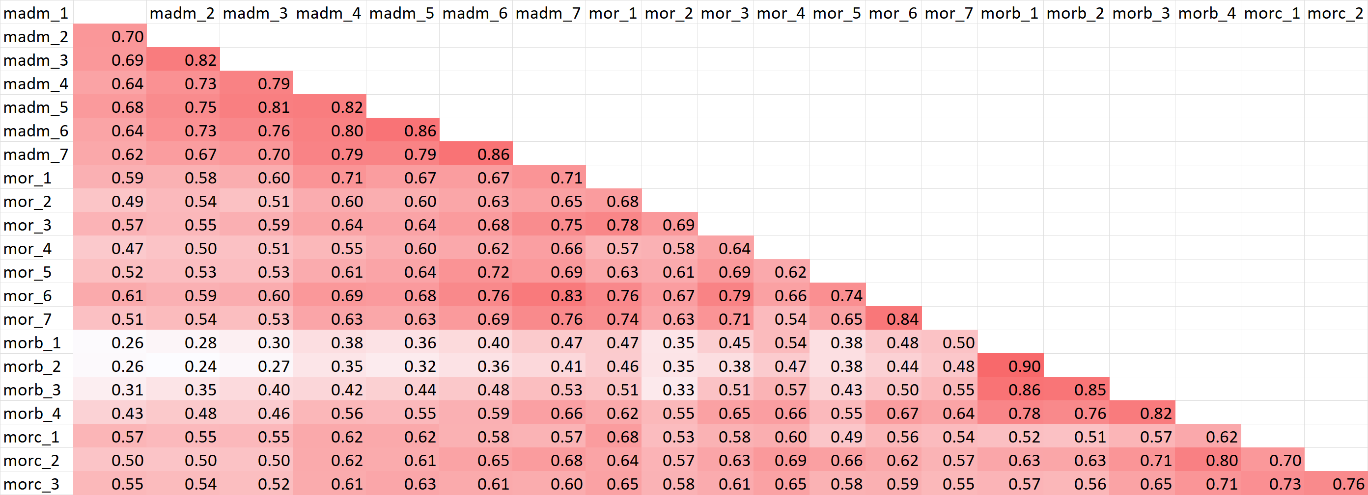


Two item sets have very strong correlation ($r\geq.85$) (madm_5 – madm_6 – madm_7 & morb_1 – morb_2 – morb_3) indicating madm_5 and morb_2 may not provide unique information.

**E.** Parallel Analysis after PCA on 21 instrument items


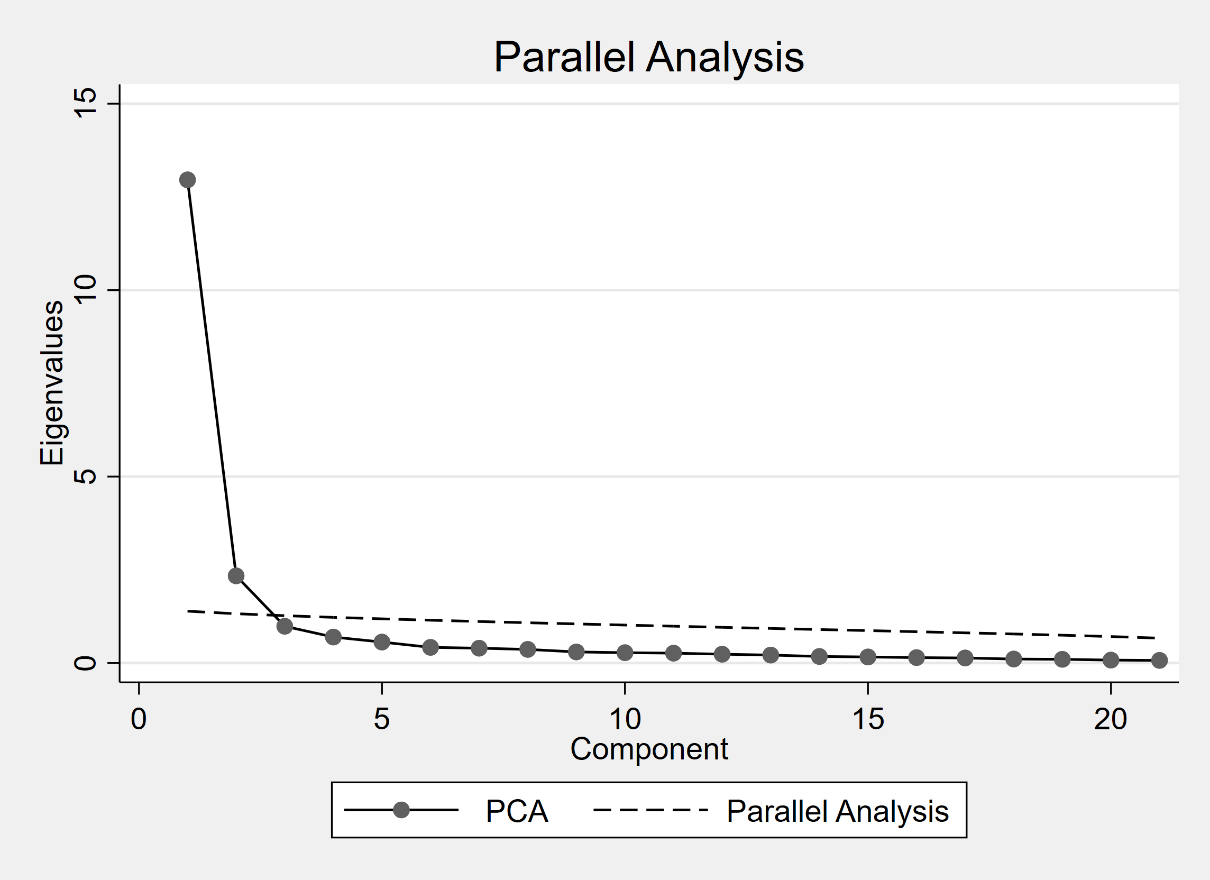


**F.** Factor Loadings and domain interpretation

| Factor | Item | Variable | Factor1 | Factor2 | Factor3 | Uniqueness |
| --- | --- | --- | --- | --- | --- | --- |
| Trust | Provider respected my personal preferences* | mor_6 | 0.8736 | 0.0678 | 0.0053 | 0.1436 |
| Trust | Provider respected my cultural preferences* | mor_7 | 0.8345 | -0.0286 | 0.0902 | 0.2409 |
| Trust | Comfortable accepting options for care recommended by provider* | mor_3 | 0.8311 | 0.0588 | 0.0222 | 0.2138 |
| Trust | Comfortable declining care offered by provider* | mor_2 | 0.8127 | 0.0652 | -0.0734 | 0.3315 |
| Trust | I chose the care options I received* | mor_5 | 0.8034 | 0.0657 | -0.0262 | 0.3025 |
| Trust | Comfortable asking my provider questions* | mor_1 | 0.6382 | 0.2017 | 0.1021 | 0.2644 |
| Trust | Provider respected my choices* | madm_7 | 0.5914 | 0.367 | 0.0287 | 0.1812 |
| Trust | Felt pushed into accepting options for care suggested by provider | mor_4 | 0.4245 | 0.148 | 0.3305 | 0.3865 |
| Informed Choice | Provider explained advantages and disadvantages of options* | madm_3 | -0.0618 | 0.983 | -0.0469 | 0.154 |
| Informed Choice | Provider told me there are different options for maternity care* | madm_2 | -0.0275 | 0.9445 | -0.0678 | 0.1989 |
| Informed Choice | Provider asked how involved in decision-making I wanted to be* | madm_1 | 0.0618 | 0.8119 | -0.0678 | 0.3208 |
| Informed Choice | I was given enough time to thoroughly consider the different care options* | madm_5 | 0.1889 | 0.7832 | -0.0181 | 0.1618 |
| Informed Choice | Provider helped me understand all information* | madm_4 | 0.2275 | 0.7224 | -0.0072 | 0.2039 |
| Informed Choice | I was able to choose what I considered best care option* | madm_6 | 0.4056 | 0.5873 | -0.0227 | 0.1814 |
| Informed Choice | Held back from asking questions because provider seemed rushed | morc_1 | -0.0064 | 0.494 | 0.4455 | 0.3512 |
| Identity | Felt treated poorly by provider because of sexual orientation, gender identity | morb_2 | -0.0599 | -0.1351 | 1.0207 | 0.1319 |
| Identity | Felt treated poorly by provider because of race, ethnicity, culture | morb_1 | -0.0199 | -0.1186 | 0.9954 | 0.1298 |
| Identity | Felt treated poorly by provider because of type of health insurance | morb_3 | -0.0635 | 0.0175 | 0.9679 | 0.1171 |
| Identity | Felt treated poorly by provider because of difference of opinion on care | morb_4 | 0.2603 | 0.0187 | 0.7307 | 0.1516 |
| Identity | Held back from asking questions because I wanted care that differed from provider recommendation | morc_2 | 0.2454 | 0.1907 | 0.5673 | 0.2455 |
| Identity | Held back from asking questions because provider might think I was being difficult | morc_3 | 0.1029 | 0.3666 | 0.5053 | 0.305 |

*Reverse scored item, inverted prior to analysis

**G.** Distribution of responses to instrument items

| N=484 | Summary |  |  |
| --- | --- | --- | --- |
|  | n (%) |  |  |
| **My provider asked me how involved in decision making I wanted to be** | | | madm_1 |
| Completely disagree | 35 (7.2%) | **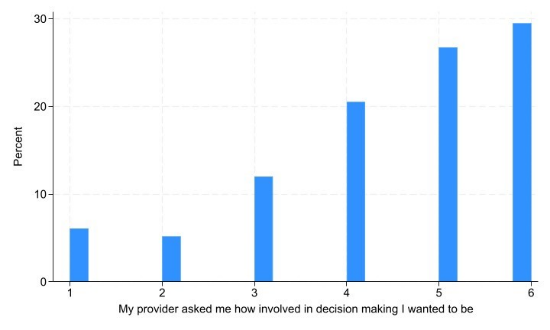** |  |
| Strongly disagree | 28 (5.8%) |  |  |
| Somewhat disagree | 57 (11.8%) |  |  |
| Somewhat agree | 88 (18.2%) |  |  |
| Strongly agree | 116 (24.0%) |  |  |
| Completely agree | 160 (33.1%) |  |  |
| **My provider told me that there are different options for my maternity care** | | | madm_2 |
| Completely disagree | 23 (4.8%) | **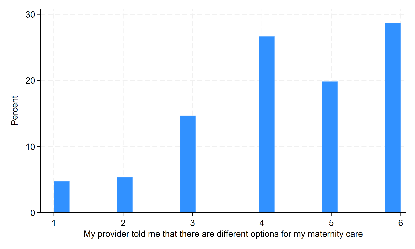** |  |
| Strongly disagree | 26 (5.4%) |  |  |
| Somewhat disagree | 71 (14.7%) |  |  |
| Somewhat agree | 129 (26.7%) |  |  |
| Strongly agree | 96 (19.8%) |  |  |
| Completely agree | 139 (28.7%) |  |  |
| **My provider explained the advantages/disadvantages of the maternity care options** | | | madm_3 |
| Completely disagree | 17 (3.5%) | **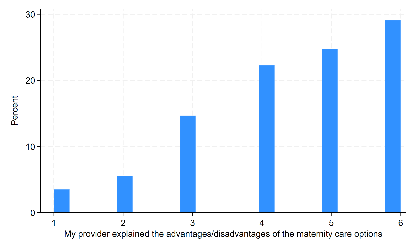** |  |
| Strongly disagree | 27 (5.6%) |  |  |
| Somewhat disagree | 71 (14.7%) |  |  |
| Somewhat agree | 108 (22.3%) |  |  |
| Strongly agree | 120 (24.8%) |  |  |
| Completely agree | 141 (29.1%) |  |  |
| **My provider helped me understand all the information** | | | madm_4 |
| Completely disagree | 8 (1.7%) | **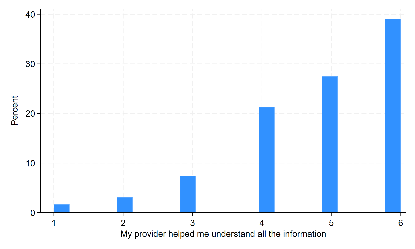** |  |
| Strongly disagree | 15 (3.1%) |  |  |
| Somewhat disagree | 36 (7.4%) |  |  |
| Somewhat agree | 103 (21.3%) |  |  |
| Strongly agree | 133 (27.5%) |  |  |
| Completely agree | 189 (39.0%) |  |  |
| **I was given enough time to thoroughly consider the different care options** | | | madm_5 |
| Completely disagree | 12 (2.5%) | **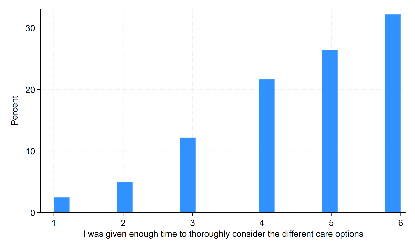** |  |
| Strongly disagree | 24 (5.0%) |  |  |
| Somewhat disagree | 59 (12.2%) |  |  |
| Somewhat agree | 105 (21.7%) |  |  |
| Strongly agree | 128 (26.4%) |  |  |
| Completely agree | 156 (32.2%) |  |  |
| **I was able to choose what I considered to be the best care options** | | | madm_6 |
| Completely disagree | 11 (2.3%) | **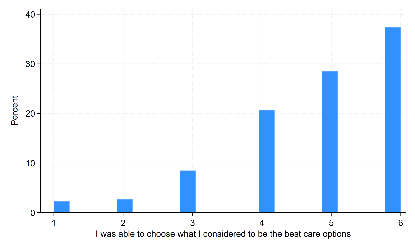** |  |
| Strongly disagree | 13 (2.7%) |  |  |
| Somewhat disagree | 41 (8.5%) |  |  |
| Somewhat agree | 100 (20.7%) |  |  |
| Strongly agree | 138 (28.5%) |  |  |
| Completely agree | 181 (37.4%) |  |  |
| **My provider respected my choices** | | | madm_7 |
| Completely disagree | 6 (1.2%) | **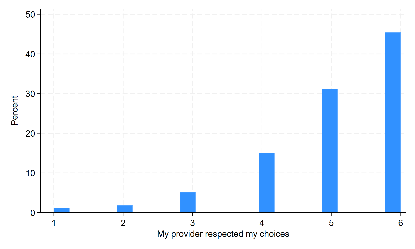** |  |
| Strongly disagree | 9 (1.9%) |  |  |
| Somewhat disagree | 25 (5.2%) |  |  |
| Somewhat agree | 73 (15.1%) |  |  |
| Strongly agree | 151 (31.2%) |  |  |
| Completely agree | 220 (45.5%) |  |  |
| **I felt comfortable asking my provider questions** | | | mor_1 |
| Strongly disagree | 2 (0.4%) | **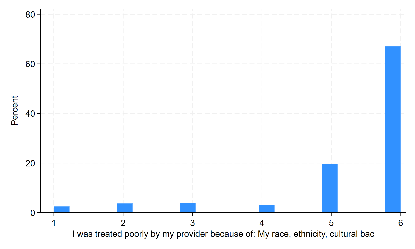** |  |
| Disagree | 4 (0.8%) |  |  |
| Somewhat disagree | 7 (1.4%) |  |  |
| Somewhat agree | 43 (8.9%) |  |  |
| Agree | 145 (30.0%) |  |  |
| Strongly agree | 283 (58.5%) |  |  |
| **I felt comfortable declining care that was offered by my provider** | | | mor_2 |
| Strongly disagree | 8 (1.7%) | **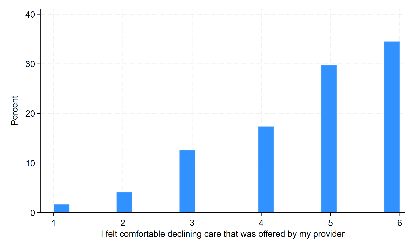** |  |
| Disagree | 20 (4.1%) |  |  |
| Somewhat disagree | 61 (12.6%) |  |  |
| Somewhat agree | 84 (17.4%) |  |  |
| Agree | 144 (29.8%) |  |  |
| Strongly agree | 167 (34.5%) |  |  |
| **I felt comfortable accepting the options for care that my provider recommended** | | | mor_3 |
| Strongly disagree | 5 (1.0%) | **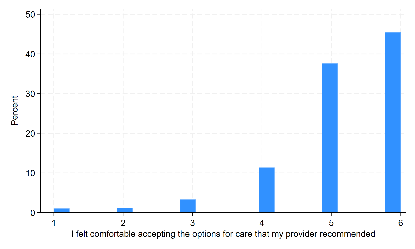** |  |
| Disagree | 6 (1.2%) |  |  |
| Somewhat disagree | 16 (3.3%) |  |  |
| Somewhat agree | 55 (11.4%) |  |  |
| Agree | 182 (37.6%) |  |  |
| Strongly agree | 220 (45.5%) |  |  |
| **I felt pushed into accepting the options for care my provider suggested** | | | mor_4 |
| Strongly agree | 23 (4.8%) | **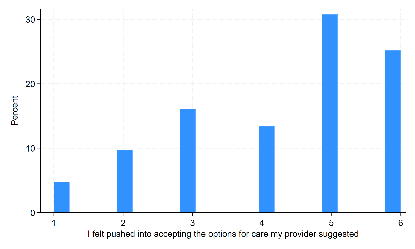** |  |
| Agree | 47 (9.7%) |  |  |
| Somewhat agree | 78 (16.1%) |  |  |
| Somewhat disagree | 65 (13.4%) |  |  |
| Disagree | 149 (30.8%) |  |  |
| Strongly disagree | 122 (25.2%) |  |  |
| **I chose the care options that I received** | |  | mor_5 |
| Strongly disagree | 3 (0.6%) | **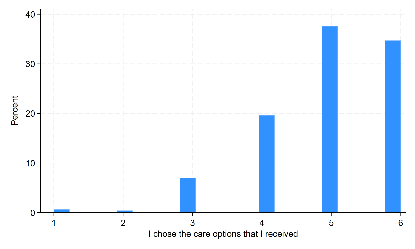** |  |
| Disagree | 2 (0.4%) |  |  |
| Somewhat disagree | 34 (7.0%) |  |  |
| Somewhat agree | 95 (19.6%) |  |  |
| Agree | 182 (37.6%) |  |  |
| Strongly agree | 168 (34.7%) |  |  |
| **My provider respected my personal references** | |  | mor_6 |
| Strongly disagree | 2 (0.4%) | **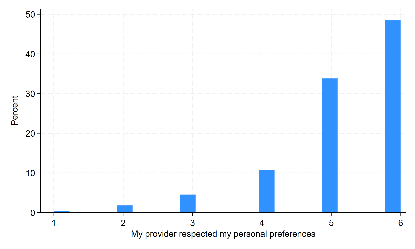** |  |
| Disagree | 9 (1.9%) |  |  |
| Somewhat disagree | 22 (4.5%) |  |  |
| Somewhat agree | 52 (10.7%) |  |  |
| Agree | 164 (33.9%) |  |  |
| Strongly agree | 235 (48.6%) |  |  |
| **My provider respected my cultural references** | |  | mor_7 |
| Strongly disagree | 2 (0.4%) | **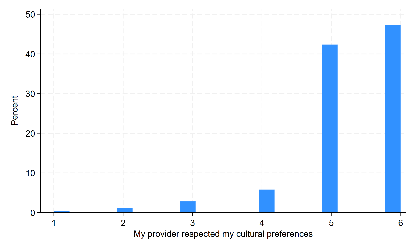** |  |
| Disagree | 6 (1.2%) |  |  |
| Somewhat disagree | 14 (2.9%) |  |  |
| Somewhat agree | 28 (5.8%) |  |  |
| Agree | 205 (42.4%) |  |  |
| Strongly agree | 229 (47.3%) |  |  |
| **I was treated poorly by my provider because of: My race, ethnicity, cultural bac** | | | morb_1 |
| Strongly agree | 12 (2.5%) | **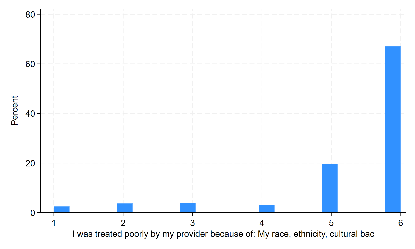** |  |
| Agree | 18 (3.7%) |  |  |
| Somewhat agree | 19 (3.9%) |  |  |
| Somewhat disagree | 15 (3.1%) |  |  |
| Disagree | 95 (19.6%) |  |  |
| Strongly disagree | 325 (67.1%) |  |  |
| **I was treated poorly by my provider because of: My sexual orientation and/or gen** | | | mob_2 |
| Strongly agree | 8 (1.7%) | **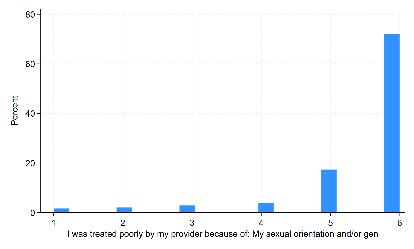** |  |
| Agree | 10 (2.1%) |  |  |
| Somewhat agree | 14 (2.9%) |  |  |
| Somewhat disagree | 19 (3.9%) |  |  |
| Disagree | 84 (17.4%) |  |  |
| Strongly disagree | 349 (72.1%) |  |  |
| **I was treated poorly by my provider because of: My type of health insurance or l** | | | morb_3 |
| Strongly agree | 11 (2.3%) | **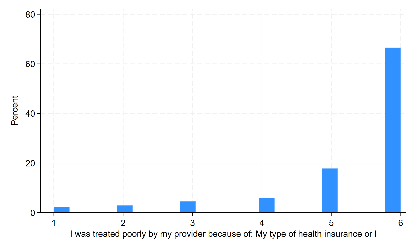** |  |
| Agree | 14 (2.9%) |  |  |
| Somewhat agree | 22 (4.5%) |  |  |
| Somewhat disagree | 29 (6.0%) |  |  |
| Disagree | 86 (17.8%) |  |  |
| Strongly disagree | 322 (66.5%) |  |  |
| **I was treated poorly by my provider because of: A difference of opinion with my** | | | morb_4 |
| Strongly agree | 10 (2.1%) | **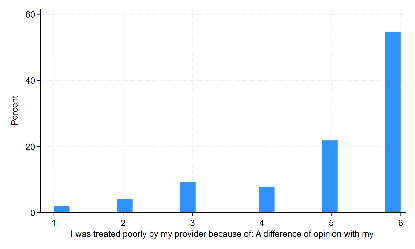** |  |
| Agree | 20 (4.1%) |  |  |
| Somewhat agree | 45 (9.3%) |  |  |
| Somewhat disagree | 38 (7.9%) |  |  |
| Disagree | 106 (21.9%) |  |  |
| Strongly disagree | 265 (54.8%) |  |  |
| **My provider seemed rushed** |  |  | morc_1 |
| Strongly agree | 33 (6.8%) | **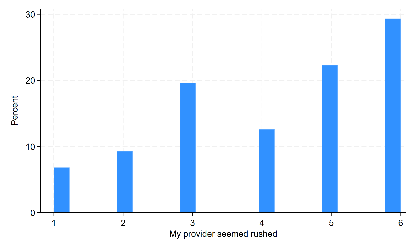** |  |
| Agree | 45 (9.3%) |  |  |
| Somewhat agree | 95 (19.6%) |  |  |
| Somewhat disagree | 61 (12.6%) |  |  |
| Disagree | 108 (22.3%) |  |  |
| Strongly disagree | 142 (29.3%) |  |  |
| **I wanted maternity care that differed from what my provider recommended** | | | morc_2 |
| Strongly agree | 8 (1.7%) | **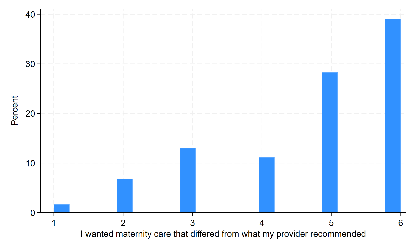** |  |
| Agree | 33 (6.8%) |  |  |
| Somewhat agree | 63 (13.0%) |  |  |
| Somewhat disagree | 54 (11.2%) |  |  |
| Disagree | 137 (28.3%) |  |  |
| Strongly disagree | 189 (39.0%) |  |  |
| **I thought my provider might think I was being difficult** | | | morc_3 |
| Strongly agree | 19 (3.9%) | **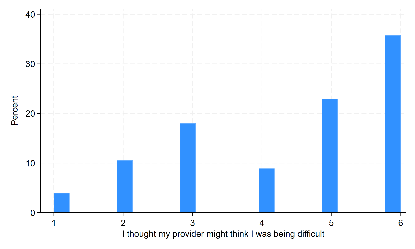** |  |
| Agree | 51 (10.5%) |  |  |
| Somewhat agree | 87 (18.0%) |  |  |
| Somewhat disagree | 43 (8.9%) |  |  |
| Disagree | 111 (22.9%) |  |  |
| Strongly disagree | 173 (35.7%) |  |  |
|  |  |  |  |
|  |  |  |  |
