# Supplementary material for: Patient-centered respectful maternity care: a factor analysis contextualizing marginalized identities, trust, and informed choice
Source: BMC Pregnancy Childbirth. 2024 Apr 11;24:267. doi: 10.1186/s12884-024-06491-2 (PMC11010273; doi:10.1186/s12884-024-06491-2)
Supplement: Supplementary file 2 — Supplementary Material 2 [file 12884_2024_6491_MOESM2_ESM.pdf]

Eligibility

1 Are you currently pregnant and living in Montana, or have you had a pregnancy in Montana in the last 5 years?

◯ Yes
◯ No
◯ Unsure

Thank you for taking the time to participate in our survey! Your responses indicate that you are not currently eligible to complete the survey. Please follow the link below to exit.

Exit survey

2 How many pregnancies have you had?

◯ 0 (currently pregnant)
◯ 1
◯ 2
◯ 3
◯ 4
◯ 5
◯ 6
◯ 7+

3 Which of the following providers were involved in your care during and/or in the 12 months following your most recent pregnancy? Please select all that apply.

☐ Family Doctor/Primary Care Provider (PCP)
☐ Certified Nurse Midwife
☐ Direct Entry Midwife / Lay Midwife
☐ Nurse Practitioner (NP)
☐ Obstetrician/Gynecologist (OB/GYN)
☐ Maternal-Fetal Medicine (MFM) Specialist/Perinatologist
☐ Physician Assistant/Physician Associate (PA)
☐ Other (please specify): __________

3a Please describe "other:"

____

4 What other clinical and community supports were involved in your care during and/or in the 12 months following your most recent pregnancy? Please select all that apply.

☐ Community Elder or Cultural Person
☐ Family Member
☐ Friend
☐ Partner
☐ Spiritual/Religious Guide
☐ Doula
☐ Nurse (LPN, RN)
☐ Mental health professional (psychologist, social worker, counselor)
☐ Other (please specify): __________

4a Please describe "other:"

____

5 If you have given birth within the last 5 years, where did you give birth? (If you have had multiple births, please consider your most recent birth).

◯ Hospital
◯ At home
◯ Birth center
◯ Other (please specify):_______
◯ I did not deliver (early pregnancy loss, miscarriage, termination)

5b Please describe "other:"

____

5 If you are currently pregnant, where do you plan to give birth?

◯ Hospital
◯ At home
◯ Birth center
◯ Other (please specify):_______

5a Please describe "other:"

____

Annual Wellness Visit

6 Did you have an annual wellness visit* the year before your pregnancy? (If you have had multiple pregnancies, please consider your most recent pregnancy).

*An annual wellness visit is a routine preventative care and counseling visit focused on maintaining a healthy lifestyle and minimizing health risks. These visits often involve a physical exam, screening tests, a discussion of health habits and health history, and education/counseling to help you make informed health decisions.

◯ Yes
◯ No
◯ Unsure

6a Please select all of the services that were included in the wellness visit:

☐ Discussion of health history (medications; allergies; substance use; and medical, surgical, family, social, and gynecologic history, including questions on reproductive, sexual, and mental health).
☐ Physical exam (height, weight, blood pressure, breast, and pelvic exam)
☐ Health screening (sexually transmitted infections (STIs), cervical cancer, breast cancer, mental health)
☐ Reproductive life planning (prepregnancy counseling, contraceptive options)
☐ Other (please specify):_______

6b Please describe "other:"

____

6c Please select all of the health screenings included in your visit

☐ Sexually transmitted infections (STIs)
☐ Cervical cancer (pap-test)
☐ Depression and/or anxiety
☐ Substance use
☐ Breast cancer (mammogram)
☐ Other screenings (please specify)

6d Please list other health screenings

____

6e Were you satisfied with the care you received at your wellness visit? (e.g., all questions/concerns addressed, information/resources provided, experience with your provider)

◯ Yes
◯ No

6f How could the wellness visit have been improved?

7 Do you have a wellness visit every year?

*An annual wellness visit is a routine preventative care and counseling visit focused on maintaining a healthy lifestyle and minimizing health risks. These visits often involve a physical exam, screening tests, a discussion of health habits and health history, and education/counseling to help you make informed health decisions.

◯ Yes
◯ No
◯ Unsure

7a Please select all the services that were included in your most recent wellness visit:

☐ Discussion of health history (medications; allergies; substance use; and medical, surgical, family, social, and gynecologic history, including questions on reproductive, sexual, and mental health).
☐ Physical exam (height, weight, blood pressure, breast, and pelvic exam)
☐ Health screening (sexually transmitted infections (STIs), cervical cancer, breast cancer, mental health)
☐ Reproductive life planning (prepregnancy counseling, contraceptive options, birth spacing)
☐ Other (please specify):_______

7b Please describe "other:"

____

7c Please select all of the health screenings included in your visit

☐ Sexually transmitted infections (STIs)
☐ Cervical cancer (pap-test)
☐ Depression and/or anxiety
☐ Substance use
☐ Breast cancer (mammogram)
☐ Other screenings (please specify)

7d Please list other health screenings

____

7e Were you satisfied with the care you received at your wellness visit? (e.g., all questions/concerns addressed, information/resources, experience with your provider)

◯ Yes
◯ No

7f How could the wellness visit have been improved?

8 In this next section we'll ask for information about your discussions with your doctor, midwife, or other care provider about your options for care (for example: prenatal testing, starting your labor, medications, where to give birth, newborn care, whether to have a cesarean, etc.). Please consider your most recent pregnancy and/or birth.

My answers describe my conversations or experiences with a:

please select the provider most involved in your perinatal care

◯ Family Doctor
◯ Obstetrician/OB-GYN Doctor
◯ Certified Nurse Midwife
◯ Direct Entry Midwife/Lay Midwife
◯ Not applicable, did not have a doctor or midwife
◯ Other

8a Please describe "other:"

____

9 Overall while making decisions about my pregnancy or birth care: I felt comfortable asking my provider questions.

◯ Strongly Agree
◯ Agree
◯ Somewhat Agree
◯ Somewhat Disagree
◯ Disagree
◯ Strongly Disagree

10 Overall while making decisions about my pregnancy or birth care: I felt comfortable declining care that was offered by my provider.

◯ Strongly Agree
◯ Agree
◯ Somewhat Agree
◯ Somewhat Disagree
◯ Disagree
◯ Strongly Disagree

11 Overall while making decisions about my pregnancy or birth care: I felt comfortable accepting the options for care that my provider recommended.

◯ Strongly Agree
◯ Agree
◯ Somewhat Agree
◯ Somewhat Disagree
◯ Disagree
◯ Strongly Disagree

12 Overall while making decisions about my pregnancy or birth care: I felt pushed into accepting the options for care my provider suggested.

◯ Strongly Agree
◯ Agree
◯ Somewhat Agree
◯ Somewhat Disagree
◯ Disagree
◯ Strongly Disagree

13 Overall while making decisions about my pregnancy or birth care: I chose the care options that I received.

◯ Strongly Agree
◯ Agree
◯ Somewhat Agree
◯ Somewhat Disagree
◯ Disagree
◯ Strongly Disagree

14 Overall while making decisions about my pregnancy or birth care: My provider respected my personal preferences.

◯ Strongly Agree
◯ Agree
◯ Somewhat Agree
◯ Somewhat Disagree
◯ Disagree
◯ Strongly Disagree

15 Overall while making decisions about my pregnancy or birth care: My provider respected my cultural preferences.

◯ Strongly Agree
◯ Agree
◯ Somewhat Agree
◯ Somewhat Disagree
◯ Disagree
◯ Strongly Disagree

Interactions with the Care Team

16 During my pregnancy, I felt that I was treated poorly by my provider because of: My race, ethnicity, cultural background, or language.

◯ Strongly Agree
◯ Agree
◯ Somewhat Agree
◯ Somewhat Disagree
◯ Disagree
◯ Strongly Disagree

17 During my pregnancy, I felt that I was treated poorly by my provider because of: My sexual orientation and/or gender identity.

◯ Strongly Agree
◯ Agree
◯ Somewhat Agree
◯ Somewhat Disagree
◯ Disagree
◯ Strongly Disagree

18 During my pregnancy, I felt that I was treated poorly by my provider because of: My type of health insurance or lack of insurance.

◯ Strongly Agree
◯ Agree
◯ Somewhat Agree
◯ Somewhat Disagree
◯ Disagree
◯ Strongly Disagree

19 During my pregnancy, I felt that I was treated poorly by my provider because of: A difference of opinion with my caregivers about the right care for myself or my baby.

◯ Strongly Agree
◯ Agree
◯ Somewhat Agree
◯ Somewhat Disagree
◯ Disagree
◯ Strongly Disagree

20 During my pregnancy, I held back from asking questions or discussing my concerns because: My provider seemed rushed.

◯ Strongly Agree
◯ Agree
◯ Somewhat Agree
◯ Somewhat Disagree
◯ Disagree
◯ Strongly Disagree

21 During my pregnancy, I held back from asking questions or discussing my concerns because: I wanted maternity care that differed from what my provider recommended.

◯ Strongly Agree
◯ Agree
◯ Somewhat Agree
◯ Somewhat Disagree
◯ Disagree
◯ Strongly Disagree

22 During my pregnancy, I held back from asking questions or discussing my concerns because: I thought my provider might think I was being difficult.

◯ Strongly Agree
◯ Agree
◯ Somewhat Agree
◯ Somewhat Disagree
◯ Disagree
◯ Strongly Disagree

Is there anything else you want to share about your interactions with your care team during your pregnancy and/or birth?

Decision Making

23 Please describe experiences with decision making during your most recent pregnancy and/or birth: My provider asked me how involved in decision making I wanted to be.

◯ Completely Agree
◯ Strongly Agree
◯ Somewhat Agree
◯ Somewhat Disagree
◯ Strongly Disagree
◯ Completely Disagree

24 Please describe experiences with decision making during your most recent pregnancy and/or birth: My provider told me that there are different options for my maternity care.

◯ Completely Agree
◯ Strongly Agree
◯ Somewhat Agree
◯ Somewhat Disagree
◯ Strongly Disagree
◯ Completely Disagree

25 Please describe experiences with decision making during your most recent pregnancy and/or birth: My provider explained the advantages/disadvantages of the maternity care options.

◯ Completely Agree
◯ Strongly Agree
◯ Somewhat Agree
◯ Somewhat Disagree
◯ Strongly Disagree
◯ Completely Disagree

26 Please describe experiences with decision making during your most recent pregnancy and/or birth: My provider helped me understand all the information.

◯ Completely Agree
◯ Strongly Agree
◯ Somewhat Agree
◯ Somewhat Disagree
◯ Strongly Disagree
◯ Completely Disagree

27 Please describe experiences with decision making during your most recent pregnancy and/or birth: I was given enough time to thoroughly consider the different care options.

◯ Completely Agree
◯ Strongly Agree
◯ Somewhat Agree
◯ Somewhat Disagree
◯ Strongly Disagree
◯ Completely Disagree

28 Please describe experiences with decision making during your most recent pregnancy and/or birth: I was able to choose what I considered to be the best care options.

◯ Completely Agree
◯ Strongly Agree
◯ Somewhat Agree
◯ Somewhat Disagree
◯ Strongly Disagree
◯ Completely Disagree

29 Please describe experiences with decision making during your most recent pregnancy and/or birth: My provider respected my choices.

◯ Completely Agree
◯ Strongly Agree
◯ Somewhat Agree
◯ Somewhat Disagree
◯ Strongly Disagree
◯ Completely Disagree

Is there anything else you want to share about your experiences with decision making during your pregnancy and/or birth?

30 In the last 12 months, did you ever eat less than you felt you should because there wasn't enough money for food?

◯ Yes
◯ No

31 In the last 12 months, has the electric, gas, oil, or water company threatened to shut off services in your home?

◯ Yes
◯ No

32 Are you worried that in the next 2 months, you may not have stable housing?

◯ Yes
◯ No

33 Do problems getting child care make it difficult for you to work or study?

(leave blank if you do not have children)

◯ Yes
◯ No

34 In the last 12 months, have you needed to see a doctor, but could not because of cost?

◯ Yes
◯ No

35 In the last 12 months, have you ever had to go without health care because you didn't have a way to get there?

◯ Yes
◯ No

36 Do you ever need help reading hospital materials?

◯ Yes
◯ No

37 Do you often feel that you lack companionship?

◯ Yes
◯ No

About You

38 What is your race and/or ethnicity? Please check all that apply.

☐ American Indian, Native American, Alaska Native
☐ African, African American, or Black
☐ Asian or Asian American
☐ Hispanic/Latinx
☐ Middle Eastern or North African
☐ Native Hawaiian or Pacific Islander
☐ White
☐ Something else, please describe: ______________
☐ Prefer not to answer

38a Please describe "something else:"

____

---

39 Do you think of yourself as:

☐ Genderqueer/gender-nonconforming neither exclusively male nor female
☐ Man
☐ Transgender man/trans man
☐ Transgender woman/trans woman
☐ Two-Spirit
☐ Woman
☐ Something else, please describe: ______________
☐ Prefer not to answer

39a Please describe "something else:"

____

---

40 What is your age in years?

◯ 18 - 29
◯ 30 - 39
◯ 40 - 49
◯ 50 - 59
◯ 60 or older

---

41 What is the highest level of education you have completed?

◯ Some high school
◯ High school diploma
◯ General Equivalency Diploma (GED)
◯ Some trade school course work completed
◯ Technical or occupational certificate
◯ Some college
◯ Associate's Degree (2 year)
◯ Bachelor's Degree (4 year)
◯ Master's Degree
◯ Doctorate
◯ Professional Degree (e.g., JD, MD, DO)
◯ Other (please specify): __________

41a Please describe "other:"

____

42 What is your current employment status?

☐ Employed Full Time
☐ Employed Part Time
☐ Self-Employed
☐ Home Maker
☐ Student
☐ Unemployed and actively seeking job
☐ Unemployed and not currently seeking job
☐ Retired
☐ Unable to work/receiving disability assistance
☐ Other, please describe: __________

42a Please describe "other:"

____

43 Have you or your child's other parent ever been incarcerated?

◯ Yes, I have
◯ Yes, my child's second parent has
◯ Yes, my child's second parent and I have both been incarcerated
◯ No

44 What is your approximate annual household/family income?

◯ $0-24,999
◯ $25,000-49,999
◯ $50,000-74,999
◯ $75,000-99,999
◯ >$100,000

45 Are you deaf, or do you have serious difficulty hearing?

◯ Yes
◯ No

46 Are you blind, or do you have serious difficulty seeing, even when wearing glasses?

◯ Yes
◯ No

47 Because of a physical, mental, or emotional condition, do you have serious difficulty concentrating, remembering, or making decisions?

◯ Yes
◯ No

48 Do you have serious difficulty walking or climbing stairs?

◯ Yes
◯ No

49 Do you have difficulty dressing or bathing?

◯ Yes
◯ No

50 Because of a physical, mental, or emotional condition, do you have difficulty doing errands alone such as visiting a doctor's office or shopping?

◯ Yes
◯ No

51 What is the zip code of your current residence?

____
